# News Items

**Published:** 1916-10

**Authors:** 


### News Items.

Special attention is called to the advertisement of the Lakeside Sanitarium which appears in this issue of the JOURNAL.

Dr. F. S. White has recently become identified with Dr. Dorbandt in the Lakeside Sanitarium, and they are prepared to care for and give personal attention to all cases of nervous and mental diseases, alcoholic and drug addictions, pellagra, etc.

The Lakeside Sanitarium is splendidly located in the suburbs of San Antonio, overlooking West End Lake. The location is ideally quiet, and the surroundings are very pleasant.

Dr. White has seen and treated a large number of cases of pellagra and knows this disease to be very curable if given early and proper treatment. In his experience of over fifteen years in the asylums of Texas he has had the opportunity of becoming so thoroughly conversant with these types of diseases that he is prepared to give his patients the benefit of the best treatment known.

---

Dr. L. B. Bibb and Dr. C. E. Gilliland, who have been connected with the Austin Sanitarium for a number of years, are in the North, where they will do post-graduate work for the coming year. Dr. Bibb will do work in the Rockefeller Institute in New York. Dr. Gilliland will pursue his studies in the St. Louis University. Both of these men are young, ambitious and progressive. They will be missed in medical circles, but we predict for them a brilliant future, and congratulate the Austin Sanitarium upon having such men connected with it.

---

To those who so graciously returned to us the August issue of the TEXAS MEDICAL JOURNAL, we hereby acknowledge our indebtedness and proffer our thanks.

---

The State Board of Medical Examiners will hold their fall examination at the Metropolitan Hotel in Fort Worth, Texas, November 14, 15 and 16.

---

Dr. G. A. Deason, formerly of Kilgore, has moved to Henderson and formed a partnership with Dr. J. A. Spivey.

---

Dr. C. S. Venable of San Antonio is in the East taking a post-graduate course.

---

Dr. D. Meredith of Wichita Falls has moved to Fort Worth.

---

Doctor: "As near as I can make out, your wife seems to have experienced a sudden shock of some kind."

Husband: "I guess that's right, I got home before 12 o'clock last night."

